# Incorporating Olive By-Products in Bísaro Pig Diets: Effect on Dry-Cured Product Quality

**DOI:** 10.3390/foods13162579

**Published:** 2024-08-18

**Authors:** Ana Leite, Lia Vasconcelos, Sergio Lopez, Divanildo Outor-Monteiro, Victor Pinheiro, Sandra Rodrigues, Alfredo Teixeira

**Affiliations:** 1 Centro de Investigação de Montanha (CIMO), Instituto Politécnico de Bragança, Campus de Santa Apolónia, 5300-253 Bragança, Portugal; anaisabel.leite@ipb.pt (A.L.); lia.vasconcelos@ipb.pt (L.V.); teixeira@ipb.pt (A.T.); 2 Laboratório para a Sustentabilidade e Tecnologia em Regiões de Montanha, Instituto Politécnico de Bragança, Campus de Santa Apolónia, 5300-253 Bragança, Portugal; 3 IES Andrés de Valdelvira, 02006 Albacete, Spain; sergiologar97@gmail.com; 4 Animal Science Department, Veterinary and Animal Research Centre (CECAV), University of Trás-os-Montes e Alto Douro (UTAD), 5000-801 Vila Real, Portugal; divanildo@utad.pt (D.O.-M.); vpinheir@utad.pt (V.P.)

**Keywords:** indigenous Bísaro pig, processed meat products, olive cake, valorization

## Abstract

The objective of this study was to assess the impact of incorporating olive cake into the diet of indigenous Bísaro pigs on the quality of processed meat products. To this end, loins and “cachaços” were processed using a standardized manufacturing flowchart to produce dry-cured products. The two products were manufactured using the same formulation, ingredients, and curing process. Concerning the physicochemical composition, there were significant differences between the two products for the parameters of a_w_ (*p <* 0.001), moisture (*p <* 0.001), total fat (*p <* 0.001), protein (*p <* 0.001), and haem pigments (*p <* 0.001). The diet significantly impacted the NaCl content (*p <* 0.05). However, neither the product nor the diet affected the fractions of saturated fatty acids (SFA), monounsaturated fatty acids (MUFA), or polyunsaturated fatty acids (PUFA) (*p >* 0.05). However, a significant difference was observed for n-3 (*p <* 0.05). Adding olive cake increased these fatty acids, and the diet containing 25% centrifuged olive cake showed the highest levels for both products. Compared with the control, the diets containing olive cake had a higher content of n-3 fatty acids, resulting in a lower PUFA n-6/n-3 ratio (*p <* 0.01).

## 1. Introduction

The production of olive oil is a well-known phenomenon on a global scale, with an average output of approximately 3 million tons of olive oil per year. Of this total, 2 million tons (or over 67%) are produced by the European Union (EU). Among the EU countries, the largest share of olive production comes from Spain, Italy, Greece, and Portugal. More than half of the total produced in the European Union (66%) comes from Spain. Italy and Greece have very similar productions, with 15% and 13%, respectively. Lastly, Portugal is responsible for 5% of the total produced in the EU. Additionally, the EU is the biggest consumer and exporter of olive oil. The EU is responsible for consuming 50% of annual production, 1.5 million tons of olive oil, and exporting 570,000 tons annually [1].

As a result of the high production of olive oil, many by-products that are highly toxic to the environment are generated. Efficient management of these by-products is necessary to reduce their environmental impact and ensure economic profitability since most of these raw materials, if not disposed of correctly, result in excessively high treatment costs. According to Molina-Alcaide et al. [2], olive oil by-products can be categorized as olive leaves, olive cake, and other by-products. The olive leaves refer to a mixture of leaves and branches from pruning the olive trees and harvesting and cleaning the olives before the oil is extracted [2]. Olive cake consists of olive pulp, skin, stone, and water. The terminology for olive cake varies depending on the extraction methods, such as pressing, extracting, or centrifuging. In the case of centrifugal extraction, it is crucial to distinguish between three-phase and two-phase systems. The main difference is the quantity of oil and moisture, with the two-phase centrifuged olive cake being more efficient and environmentally friendly (with a higher moisture content and lower oil content) [3]. As far as pressed olive cake is concerned, extraction is achieved by a discontinuous press process, a more traditional extraction method. The nutritional valorization of these by-products has become a key focus for various research sectors. While they are widely used in ruminant feed, their application in other animal species remains limited.

Furthermore, these by-products are an energy resource due to their lignin content, providing a high calorific value with a low ash content [4]. Another potential application for this product is composting, which can be used as a fertilizer or a component of agricultural substrates [5]. Olive cake has had various uses but remains an under-exploited resource [2]. The market for commercializing raw materials for animal feed has been significantly constrained by price volatility.

Moreover, incorporating this by-product makes perfect sense in the northern region of Trás-os-Montes (Portugal), given the amount of olive cake produced and the number of farms with Bísara animals. In addition, the time of year when these by-products are obtained coincides with the finishing phase of these animals. Therefore, transporting this by-product is more accessible due to the proximity of the farms and oil mills, and storage is also more accessible. Bísaro pigs offer products characterized by high quality [6]. Due to the characteristics of their meat, native breeds are highly valued for producing high-quality dry-cured products [7].

Consequently, the main objectives of this work are to (1) evaluate the potential of olive cake (pressed and centrifuged) as a diet component for Bísaro pigs; (2) assess effects on the physicochemical composition of the two processed products obtained on an industrial scale; (3) analyze the impact of these products on the fatty acid profile; and (4) examine the influence of cutting the Longissimus thoracis lumborum (LTL) muscle in obtaining the two processed products.

## 2. Materials and Methods

### 2.1. Experimental Diets and Slaughter Procedure

The animals used were indigenous Bísaro pigs, kept in an extensive system on the farm belonging to the company Bísaro-Salsicharia Tradicional, Lda^®^ (Gimonde-Bragança, Portugal). The first diet consisted of a traditional diet typical of these native breeds in an extensive system, thus considered the control diet. The second diet included the Base diet + 15% of centrifuged olive cake (BgCf15). The third diet consisted of a Base diet + 25% of centrifuged olive cake (BgCf25). The fourth diet consisted of a Base diet + 15% of pressed olive cake. The diets’ chemical composition and fatty acid profile are presented in Table 1.

**Figure 1:**
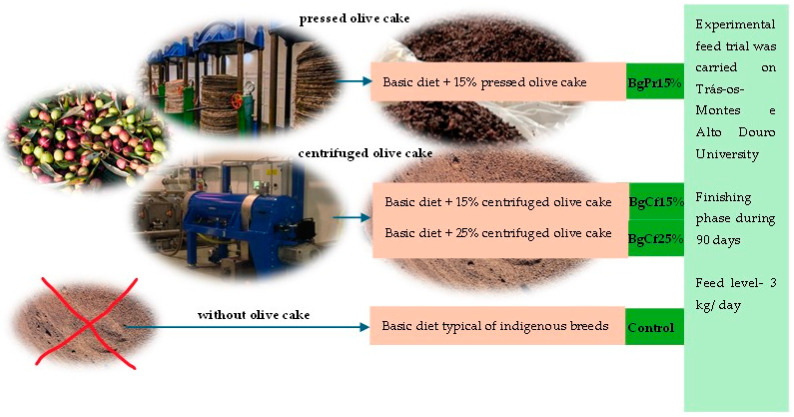
Schematic of the diets applied to the Bísaro pigs.

The Bísara animals were reared under the supervision of the University of Trás-os-Montes and Alto Douro in Vila Real, Portugal. A total of 48 animals were used for this study. The animals were separated into distinct groups to receive different diets (Figure 1). The control group was given a traditional diet for this indigenous breed, consisting of local vegetables, cereals, and feed appropriate for each growth stage. The other diets also had the same Base diet as the control diet, plus the type of olive cake with 15 and 25 percent (see Table 1). The animals were fed these diets during the finishing phase (90 days) and with an average feed of 3 kg daily. The University of Trás-os-Montes e Alto Douro team monitored the animals to ensure the diets were administered correctly throughout the finalization process. 

Once the final finishing phase was complete, the animals were slaughtered at 12 months of age, with an average live weight of approximately 135 kg. The Bragança Municipal Slaughterhouse team was responsible for the slaughter. The methodology employed for the slaughter and carcass preparation has been previously described by Álvarez-Rodríguez and Teixeira [8]. All animals were provided with appropriate care and were slaughtered following EU Council Regulation (EC) No. 1099/2009 [9], which sets out animal welfare regulations at the time of slaughter. After the slaughter and cutting, the joints obtained were transported to Bísaro-Salsicharia Tradicional, Lda^®^, which was responsible for processing them.

### 2.2. Dry-Cured Bísaro Loin and “Cachaço”

**Figure 2:**
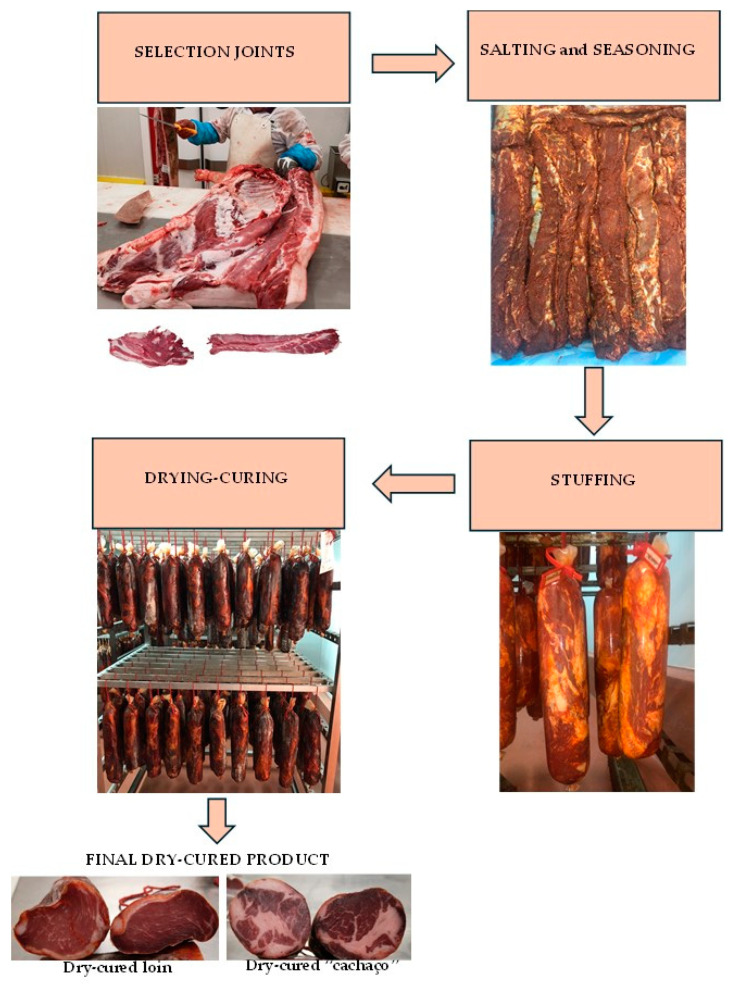
Process of obtaining Bísaro dry-cured loin and dry-cured “cachaço”.

The curing process was carried out at Bísaro-Salsicharia Tradicional, Lda^®^, using forty-eight loins and forty-eight “cachaços” from forty-eight slaughtered animals (Figure 2). The ingredients were added in decreasing order: 1.5% salt, 0.5% paprika, 0.5% garlic, and 0.1% oregano. Both the loins and “cachaços” were dry-cured for 60 days. After removing the muscles from the carcasses, the pieces were chilled in a chamber at 2–5 °C, and excess surface fat was removed from each piece. Selected pieces then underwent the curing process. In the salting and seasoning phase, the pieces were placed on a rotating drum for approximately 30 min after adding salt, paprika, garlic, and oregano. After mixing, the joints were transferred to a container and placed in a refrigeration chamber at a temperature range of 2–4 °C with approximately 90% relative humidity for 4 days to allow the ingredients to penetrate. Next, the pieces were stuffed into collagen casings. The final phase was drying and curing, during which significant biochemical changes occurred. The temperature and humidity were adjusted as the curing progressed: for the first 15 days, the cuts were kept at 4–8 °C with 80–90% relative humidity. Subsequently, the temperature was raised to 8–12 °C with 70–80% relative humidity for another 15 days. Finally, for the last 20 days, the product was maintained at 12–18 °C with 60–70% relative humidity. This curing process has been validated by the company Bísaro-Salsicharia Tradicional Lda^®^, considering all the quality and food safety standards. It should be noted that the company responsible for the entire manufacturing flowchart for this type of product has been certified for over seven years by extremely demanding quality benchmarks (IFS-Food).

### 2.3. Chemical Composition and Physicochemical Analysis of Dry-Cured Loin and Dry-Cured “Cachaço”

All the physicochemical analyses on these two products were conducted using the following Portuguese standards. Water activity was assessed according to AOAC [10] using a HigroPalm Rotronic 8303 probe (Bassersdorf, Switzerland). Moisture content was determined according to the Portuguese standard NP 1614 [11]. For this, 5 mL of ethanol (96% *v*/*v*) was added to 3 g of sample, and the samples were dried in a drying oven (Raypa DO-150, Barcelona, Spain) at 103 ± 2 °C for 24 h. Ash content was determined according to the Portuguese standard [12]. To do this, 1 mL of magnesium acetate (15% *w*/*v*) was added to 3 g of sample in crucibles. The samples were then heated to 550 ± 25 °C for 5 h in a muffle furnace (Vulcan BOX Furnace Model 3-550, Yucaipa, CA, USA). Protein content was measured following the Portuguese standard [13] using the Kjeldahl Sampler System (K370, Flawil, Switzerland) and Digest System (K-437, Flawil, Switzerland). In 25 mL of sulfuric acid (97%), two catalyst tablets and 2 g of sample were placed in mineralization tubes. Following the completion of mineralization, the distillation procedure was performed. Subsequently, the distillate was titrated using a hydrochloric acid solution, and the necessary volume was registered. The determination of hydroxyproline and collagen content was carried out by Portuguese Standards NP 1987 [14]. The haem pigment content [15] was determined by measuring the reflectance on the exposed surface using spectroscopy with a Spectronic Unicam 20 Geneys instrument. The results are expressed as mg myoglobin/g fresh muscle. Additionally, the total chloride content was assessed following the methodology specified in the Portuguese Standard NP 1845 [16], expressed as a percentage by mass of sodium chloride.

### 2.4. Fatty Acid Analysis

Fatty acids in dry-cured loin and dry-cured “cachaço” samples were analyzed at the ESA-IPB Laboratory. Total lipids were extracted from 25 g of meat using the Folch procedure [17]. The fatty acid profile was determined from 50 mg of fat. Fatty acids were transesterified following the method described by Domínguez et al. [18]. After adding 4 mL of sodium methoxide solution and vortexing intermittently for 15 min at room temperature, 5 mL of H_2_SO_4_ solution (50% in methanol) was added. Then, 2 mL of distilled water was added, followed by additional vortexing. The organic phase, containing the methyl esters of fatty acids, was extracted with 2.35 mL of hexane. The separation and quantification of fatty acid methyl esters were carried out using a gas chromatograph (GC-Shimadzu 2010Plus; Shimadzu Corporation, Kyoto, Japan) equipped with a flame ionization detector and an automatic sample injector AOC-20i and using a Supelco SP^TM^-2560 fused silica capillary column (100 m length, 0.25 mm i.d., 0.2 µm film thickness). Fatty acid contents were calculated using chromatogram peak areas and were expressed as g per 100 g of total fatty acid methyl esters. Additionally, the percentage of saturated fatty acids (ΣSFA), monounsaturated fatty acids (ΣMUFA), polyunsaturated fatty acids (ΣPUFA), the ratio PUFA n-6/n-3, and Σtrans were calculated according to Vieira et al. [19]. To assess lipid quality, the atherogenicity index (AI) and the thrombogenicity index (IT) were calculated following the Ulbricht and Southgate methods [20].

### 2.5. Statistical Analysis

The Shapiro–Wilk test was used to test data for normal distribution and homogeneity of variance. Next, the effect of diet and type of product, and the interaction between diet and product, on the physicochemical composition and fatty acid profile were examined using analysis of variance (ANOVA) with the general linear model (GLM) procedure, in which these parameters were defined as dependent variables and diet and type of product as fixed effects. The results were presented in terms of mean values and standard error of the mean (SEM). When there was a significant effect (*p <* 0.05), the means were compared using Student’s *t*-test. To extract a few key combinations (called principal components) from the group of measured variables that capture most of the variability in those variables, we conducted a principal component analysis (PCA). Each principal component was determined by linearly combining the correlation matrix’s eigenvectors. The eigenvalues indicate how much variance each component holds. Moreover, a multiple factor analysis (MFA) related to principal components analysis (PCA) was performed to produce a table of eigenvalues, summary plots, and a consensus map. All analyses were performed using the statistical package JMP^®^ Pro 17.0.0 by 2023 SAS Institute Inc.© (Cary, NC, USA).

## 3. Results and Discussion

### 3.1. Physicochemical Composition

The results of the chemical composition of dry-cured loin and dry-cured “cachaço” are presented in Table 2. This table shows the effect of the product on each of the parameters studied, the impact between the diets, and the interaction between the type of product and the diet applied. Regarding the interaction between the product and the diets, the parameters a_w_, ash, and NaCl content showed significant differences (*p <* 0.05). The other parameters (moisture, total fat, protein, collagen, and haem pigments) did not show significant results between the type of product and the diet applied. The product type significantly affected (*p <* 0.001) the parameters of a_w_, moisture, total fat, protein, and haem pigments. On the other hand, collagen, NaCl content, and ash content were not significantly influenced (*p >* 0.05) by the product type. We can also see that the diet did not affect most of the physicochemical parameters studied, except for the NaCl content.

Concerning water activity (a_w_), the values obtained for the dry-cured loin ranged from 0.896 to 0.856, while those for the dry-cured “cachaço” ranged from 0.862 to 0.815. A statistically significant correlation was observed between the product and the diet (*p <* 0.05) for the water activity parameter. The highest value for this parameter (0.896) was observed for the dry-cured loin with the 15% pressed olive cake diet (Pr15), while the lowest value for this parameter was obtained for the dry-cured “cachaço” with the same diet. The a_w_ value was significantly higher (*p <* 0.001) in all diets (including the control) for the dry-cured loin compared to the dry-cured “cachaço”. These differences in water activity values align with the results obtained for the moisture parameter. The dry-cured loin, influenced by fat content (discussed later), has a lower water activity and moisture content than other forms of dry-cured loin.

The a_w_ value for the dry-cured loin control was the highest, exceeding the values reported for the same product in a study where the olive cake was not added to the diet of the Bísaro pig [21]. In contrast, the a_w_ values observed in this work for diets containing olive cake are consistent with those reported in another study [21] (for the same product type and adding olive cake in other percentages). In previous studies, different authors reported average values of 0.841 for dry-cured Celta loin [22], 0.830 for dry-cured Korean loin [23], 0.838 for dry-cured Polish neck [24], and 0.838 for dry-cured foal loin [25]. Similar water activity values have been reported for other dry-cured products, including dry-cured shoulder [26,27,28,29]. The water activity of a food product is a significant factor that affects the safety of dry-cured meat products. It serves as a fundamental indicator of the shelf life, ensuring nutritional stability and identifying the types of microorganisms present [30]. The combination of water and salt creates osmotic changes that result in dehydration, which removes water from the meat [31]. Water can facilitate the attachment of a radical species and the removal of hydrogen from the fatty acids, thereby initiating the oxidation process [22]. Therefore, both products exhibited water activity values that align with the criteria for dry-cured products with the desired microbiological stability. Furthermore, adding olive cake to the Bísaro pig feed did not exert any discernible influence, whether positive or negative. Another crucial parameter that serves as an indicator of product ripeness is moisture content.

The moisture content of the dry-cured loin was found to be significantly higher (*p <* 0.001) than that of the dry-cured “cachaço”. The mean moisture content of the dry-cured loin ranged from 40.02 to 42.73%. Meanwhile, the dry-cured “cachaço” range was 30.99 to 33.43%. Including olive cake in the animals’ diet did not result in any observable change in moisture content. The moisture content of dry-cured products is inversely proportional to the maturation time [32]. The values obtained in this study were higher for both products than those observed by other authors [21]. Nevertheless, comparable values to those observed in the dry-cured “cachaço” have been documented in a conventional dry-cured product from Spain’s Mediterranean coast [33] and in the traditional Italian product “coppa” [34]. Similar values (42%) were obtained for Iberian dry-cured loins [34,35], as well as for other dry-cured loins [22,25,36].

The observed variations in ash content are primarily attributed to the sodium chloride content. The ash values exhibited a range of 4.25 to 7.21 for dry-cured loin and 4.97 to 6.13 for dry-cured “cachaço.” The interaction between the product and diet was statistically significant (*p <* 0.01). Nevertheless, introducing the olive cake diet or the type of product used did not result in significant differences in ash content. Other studies have reported higher ash values for dry-cured foal loin [25], “, Bísaro dry-cured shoulder [29], and Celta dry-cured ham [37]. Similar values have been observed in other studies involving Iberian dry-cured loin [38] and Turkish dried meat [39].

The total fat content exhibited a statistically significant difference (*p <* 0.001) between the dry-cured loin and dry-cured “cachaço”, with values ranging from 16.90 to 24.62% and from 36.59 to 43.37%, respectively. Concerning dietary factors, no impact was observed on the total fat content of the processed products. Although these two products originate from the same *Longissimus thoracis lumborum* (LTL) muscle, they are derived from disparate sections. The “cachaço” is obtained from the proximal region of the LTL muscle, situated in the cervical area of the column and extending to the fifth thoracic vertebra, as evidenced by its location beneath the scapula. The loin is obtained from the lumbar region of the LTL muscle. The high complexity of the meat matrix results in products derived from the same muscle exhibiting markedly disparate values for the total fat parameter.

A reduction in protein content is anticipated to accompany an elevated fat content. As demonstrated in Table 2, the protein content was significantly higher (*p <* 0.001) in the dry-cured loin, with mean values ranging between 29.73 and 34.26%. In contrast, the dry-cured “cachaço” exhibited lower protein values, ranging from 23.40% to 24.99%. The application of the olive by-product diets did not result in any discernible impact on the protein content of the products under investigation. Other studies have also observed this difference between dry-cured loin and “cachaço” [21]. A product designated as dry-cured coppa exhibited similar values to those observed in the dry-cured “cachaço” [34]. The same values have also been reported by other authors for Turkish dried meat [39], dry-cured Celta ham [37], and Bísaro dry-cured shoulder [29]. Higher values were observed in the case of the dry-cured foal [25].

No significant differences (*p >* 0.05) were observed between diets with olive cake or between the two product types concerning collagen content, which ranged from 2.83 to 3.66% and from 2.55 to 3.15% in the dry-cured loin and dry-cured “cachaço”, respectively.

The total haem pigments, expressed as myoglobin concentration, exhibited a statistically significant difference between the two products (Bísaro dry-cured loin and dry-cured “cachaço”). The myoglobin content for these products ranged from 2.15 to 2.72 mg/g and 3.84 to 4.33 mg/g in the case of the dry-cured loin and dry-cured “cachaço”, respectively. The incorporation of pressed and centrifuged olive cake did not affect this parameter, as evidenced by the findings of other researchers [21].

Significant differences were observed in the chloride content for the interaction between diet and type of product *(p <* 0.001) and for the addition of olive cake (*p <* 0.05). The sodium chloride content exhibited variability, ranging from 2.76 to 6.53% in the dry-cured loin and from 4.01 to 5.36% in the dry-cured “cachaço”. The highest salt content was observed in the dry-cured loin with the centrifuged olive cake diet at 25%. This value represents the ash content observed in the Cf25 diet in the dry-cured loin. Although a difference in the salt content of the dry-cured loin was observed, it cannot be definitively concluded that the 25% olive cake inclusion was the sole factor responsible for this value. This same pattern was not observed in the dry-cured “cachaço” case. The average NaCl value for the dry-cured loin was lower than that of the dry-cured “cachaço”. The dry-cured loin (Cf25) exhibited a markedly elevated value, resulting from a notable diet impact. It is imperative to reiterate that these products were not manufactured in a laboratory setting. Therefore, it is not possible to attribute the high levels of sodium chloride observed solely to the diet. Similar NaCl content values were observed in Salame Milano (4.3%), Coppa (5.9%), Parma Ham (6.1%) [40], and dry-cured foal “Cecina” [41].

### 3.2. Fatty Acids Composition

The fatty acid (FA) composition of the dry-cured loin and dry-cured “cachaço” is presented in Table 3 for analysis purposes. The table illustrates the impact of the product and the effect between diets on the fatty acid profile. For both products, the predominant fatty acids were oleic acid as monounsaturated fatty acid (MUFA), palmitic acid, stearic acid as saturated fatty acid (SFA), and linoleic acid as polyunsaturated fatty acid (PUFA). These four acids account for more than 93% of the total fatty acids in both products. These results align with pork’s typical fatty acid composition and are consistent with the fatty acid profile described by Leite et al. [42]. A comparable pattern in FA distribution has been documented in various types of dry-cured products [21,34,43,44,45,46,47]. Except for C18:0 fatty acid, no significant differences were observed in the remaining predominant fatty acids between the two products studied. The addition of olive cake to the animal diets had no discernible impact. Significant differences were observed between the products for the following FA: C15:0, C17:0, C18:0, C20:0, C18:3n-6, C22:0, C23:0, and n-3. As for the diet, the inclusion of olive cake in the animal diet of Bísaro pigs had a significant influence on the following FA: C16:1n-7, 9t-C18:1, C20:1n-9, C18:3n-3, C20:2n-6, n-3, and ratio n-6/n-3. The FA fractions were not affected by the type of product studied. Similarly, adding centrifuged and pressed olive cake in 15 and 25% percentages did not influence the lipid quality of dry-cured loin and dry-cured “cachaço”. Therefore, despite numerous factors that can affect the lipid composition of meat, such as diet and genetic lineage, the fatty acid profile of pork remains consistent with the typical pork profile.

Concerning MUFAs, oleic acid was the most prevalent FA, representing approximately 93% of the total MUFA content. This high proportion is significant for both products and represents an essential nutritional aspect, as this fatty acid fraction has been demonstrated to reduce cardiovascular risk factors [48]. Furthermore, MUFA has been shown to reduce plasma LDL cholesterol levels without compromising the anti-atherogenic properties of HDL cholesterol lipoproteins [49]. Oleic acid levels ranged from 51.11 to 52.15% in the dry-cured loin and from 51.15 to 51.95% in the dry-cured “cachaço”, and these levels remained unchanged with the introduction of olive cake. Although the chemical composition of these two products showed significant differences in total fat content (Table 2), there were no differences between the two products regarding oleic acid. Similar MUFA were observed in Bísaro dry-cured loin and dry-cured “cachaço” [21]. Other authors have reported higher values of this acid in Iberian ham [50], Iberian dry-cured loin [34], dry-cured “coppa” [34], and Bísaro shoulder [29]. Lower MUFA values were reported in Iberian “lacón” [51], Korean dry-cured loin [23], Italian dry-cured loin [47], and Celta dry-cured ham [43].

Regarding SFA, the predominant FA was palmitic acid, showing levels of around 63% of the total SFA. Like oleic acid, palmitic acid levels were unaffected by the product type or olive cake’s inclusion in the diet. We obtained values of between 40.52 and 41.52% for the dry-cured loin and 40.55–41.16% for the dry-cured “cachaço”. The SFA fraction was also not influenced by adding olive cake, and there were no significant differences between the two products regarding palmitic acid content. Similar SFA was observed in Bísaro dry-cured loin and dry-cured “cachaço” [21], and Iberian dry-cured loin [52]. Lower values for this fraction were obtained in Bísaro shoulder [29], Celta dry-cured loin [44], Iberian lacón [51], Iberian and Serrano Ham, Bayonne and Corsican Ham, Parma, and San Daniele Ham, Jingua Ham [49], and dry-cured “coppa” [34]. Other authors have reported higher values of SFA in Korean dry-cured loin [23] and Croatian and Montenegrin dry-cured meat [46]. In agreement with other authors [53,54] about SFA, mortality rates correlated positively with the average percentage of dietary energy from saturated fatty acids.

For PUFAs, linoleic acid was the predominant FA, accounting for more than 95% of the total PUFA content. The levels of linoleic acid were unaffected by the product type or the olive cake’s inclusion in the animal diet. Similar PUFA were observed in Bísaro dry-cured loin and dry-cured “cachaço” [21], Korean dry-cured loin [23], and Croatian and Montenegrin Prosciutto [46]. Other authors have reported higher values of PUFA in Bísaro shoulder [29], Celta dry-cured “lacón” [51], Iberian and Serrano dry-cured loin, Bayonne and Corsican Ham, Parma and San Daniele Ham, Jingua Ham [49], Pancetta and Croatian and Montenegrin dry-cured sirloin [46], and dry-cured “coppa” [34]. Lower values for this fraction were obtained in Iberian dry-cured loin [52]. It should be noted that PUFA is highly susceptible to oxidative degradation and is converted into other molecules [55]. For this reason, the curing process reduces the PUFA content in the final product. Adding a by-product such as olive cake did not counteract this reduction in unsaturated fatty acid.

Compared to previous studies’ findings [21], including a higher percentage of olive cake (centrifuged and pressed) does not influence the MUFA, SFA, and PUFA fractions.

Regarding trans fatty acids, no significant differences were observed between the two product types (*p >* 0.05), with average values of 0.21 for dry-cured loin and 0.20 for dry-cured “cachaço”. These values are below the recommended levels [56] and lower than those reported by other authors for processed Iberian dry-cured ham [57] and Bísaro shoulders [29]. Nevertheless, significantly lower values were observed in the group that consumed a diet including 25% centrifuged olive cake. Compared to the control diet, the diet containing 25% olive cake (Cf25) resulted in more favorable trans fatty acid values. In contrast, the diet with 15% centrifuged olive cake and 15% pressed olive cake exhibited detrimental outcomes concerning this category of trans fatty acid.

The PUFA/SFA and n-6/n-3 ratios, along with the IT and IA indices, are important indicators of the healthiness of fat in food. The recommended PUFA/SFA ratio for a healthy diet is 0.4 or less [58]. For the ratio n-6/n-3, the internationally recommended value for a healthy diet is 4 [59], with the optimal value being 1 [60,61,62]. Genetic modification and dietary changes have proven to be relatively effective strategies for achieving more desirable n-6/n-3 values in these products [43]. There was no significant difference in the n-6/n-3 ratio between the two products studied.

On the other hand, the diets applied with olive cake proved to be significantly different (*p <* 0.05). From the results shown in Table 3, adding olive cake to the animals’ diets significantly reduces the n-6/n-3 ratio. This trend was not observed in other studies for Bísaro dry-cured loin and dry-cured “cachaço” with 10% olive cake added [21]. Therefore, we can say that introducing a percentage of 15 and 25% olive cake in the animal diet contributes to a decrease in this ratio. Higher values of this ratio were observed in Iberian and Parma dry-cured ham [49]. However, significantly lower values were observed in Serrano dry-cured ham, Bayonne dry-cured ham, and Corsican dry-cured ham [49]. Unsaturated fats, especially PUFAs, are well known as healthy fats with critical bodily functions, such as cell growth and development and disease prevention [63]. Although linoleic acid is an essential fatty acid, it should be noted that an excessively high intake of this PUFA may not be beneficial. Studies [64,65] have shown that excessive intake of linoleic acid has a pro-inflammatory effect. Fatty acids n-6 promote vasoconstriction and the formation of blood clots, while n-3 acids have the opposite effect [66]. Among the PUFA n-3, docosahexaenoic acid (C22:6n-3, DHA) is preventive and therapeutic in some chronic inflammatory diseases [67]. In small quantities, these FAs are crucial for the correct cerebral and visual development of the fetus and the maintenance of neural and visual tissues throughout life [67]. The human body cannot synthesize this n-3 FA (DHA) type, which is only obtained through food [68]. This study revealed significant variations in the level of PUFA n-3 based on both the type of product and the diet used. The n-3 content increased with olive cake in the diet, containing 25% centrifuged olive cake, resulting in the highest n-3 values for both products. For dry-cured “cachaço”, n-3 values ranged from 0.26 to 0.33, whereas for dry-cured loin, the values were lower, ranging from 0.22 to 0.28.

A lower IA value is indicative of a reduced saturated-to-unsaturated fatty acid ratio, whereas a lower IT value is associated with a diminished risk of developing blood clots [69]. No significant differences were observed between the two product types regarding the IA and IR indices, nor was there any impact from the olive cake diets. Although no entity or organization provides reference values for these indices [70], it is generally understood that lower IA and IT values indicate better nutritional quality, potentially reducing the risk of coronary heart disease. Lower values for these indices have been reported in dry-cured “coppa” of Nero Siciliano pig [34] and Bísaro shoulder [29]. Similar values were obtained for Bísaro dry-cured loin and “cachaço” in animals fed with olive cake [21]. Other authors have reported that the intramuscular fat and backfat indices were affected by including 10% olive cake in the pig’s diet, increasing the MUFA and PUFA content and improving the IA and IT quality indices [71]. As reported by Cava et al. [72], an elevated h/H ratio indicates enhanced nutritional adequacy of the fat content in the food. In this study, the ratio h/H was not influenced by the diets applied or the product type (*p >* 0.05). The dry-cured loin obtained values between 2.00 and 2.09 and the dry-cured “cachaço” between 2.00 and 2.07. Lower values of this ratio were obtained in Bísaro dry-cured loin and “cachaço” [21]. Higher h/H values were observed in Celtic ham with a chestnut-based diet [43]. In the case of fresh pork, different cuts of meat obtained different h/H ratio values [73]. Therefore, not only the type of feed but also the joint influences this ratio. Considering the value obtained for fresh loin (2.170) [73], the curing process does not affect this nutritional index.

### 3.3. Principal Component Analysis (PCA)

**Figure 3:**
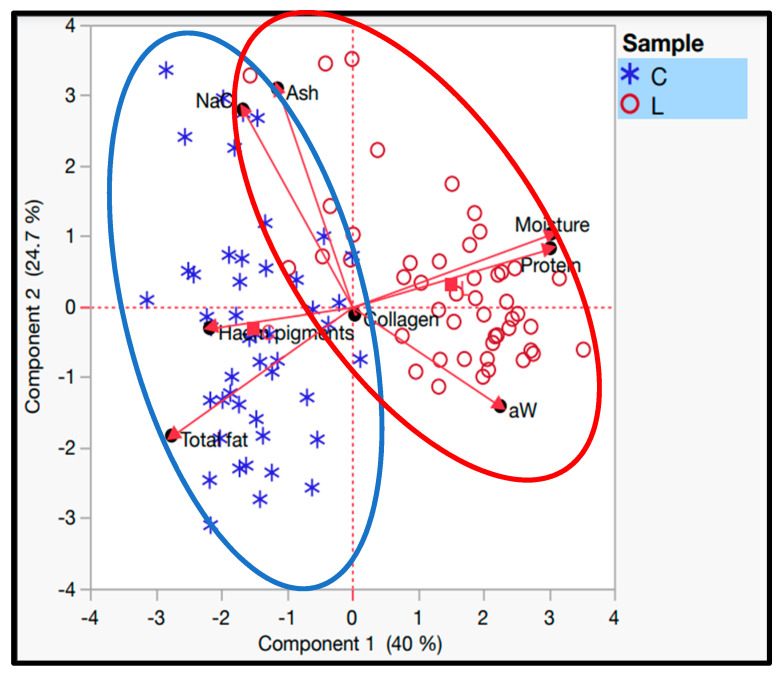
Biplot principal component analysis (C-dry-cured “cachaço”; L-dry-cured loin).

Principal component analysis (PCA) is a statistical technique used to simplify a dataset by transforming the original variables into a smaller set of new variables, called principal components, which capture the essential characteristics of the data. These principal components are linear combinations of the original variables that maximize the total variance. The primary graphical output of PCA is often a biplot, which maps the cases using the principal components and includes the original variables to help interpret the distances between case positions [74]. Figure 3 displays the results of the principal component analysis in the form of a biplot. The first principal component (*p <* 0.001) explained 40% of the total variance, and the second principal component (*p <* 0.001) explained 24.7% of the total variance. The two principal components explained the total variance of 64.7%. This principal component analysis shows a separation between the dry-cured loin and the dry-cured “cachaço”. The physicochemical characteristics that best explain the dry-cured loin are salt content, total fat, and haem pigments. On the other hand, water activity, protein content, and moisture are the factors that best explain the dry-cured loin. That said, the principal component analysis shows that although the two products (dry-cured loin and dry-cured “cachaço”) come from the same muscle (*Longissimus thoracis Lumborum),* their chemical composition is very different.

## 4. Conclusions

The results indicate that incorporating centrifuged and pressed olive cake in different percentages does not impact the physicochemical properties of dry-cured products. However, significant differences were observed between the two products, particularly in total fat, protein, a_w_, moisture, and haem pigments. Significant interactions between the product and diet were also observed for a_w_, ash, and NaCl parameters. This suggests that, despite both products originating from the same muscle (LTL), they are distinct, which can enhance their appeal to consumers. Adding olive cake significantly improved the n-3 content, with the olive cake with the highest percentage (Cf25) obtaining the highest n-3 value. These values influenced the PUFA n-6/n-3 ratio, showing the same trend, with significantly lower results in olive cake diets. There are no further differences in the different fatty acid fractions and nutritional quality indices. Still, it would be necessary for future work to understand how n-3 can influence the stability of the product and how these differences can affect the final product’s organoleptic properties and consumer acceptability.

## Figures and Tables

**Table 1:** Ingredient composition of the experimental diets (g/kg, as fed basis) and fatty acids composition (g/100 g).

	Diets			
	Control	BgCf15	BgCf25	BgPr15
**Chemical composition of the diet**				
DM	86.35	83.76	90.77	88.40
OM	94.73	95.35	95.03	95.00
NDF	20.06	27.47	36.63	27.50
ADF	7.62	12.31	23.67	13.97
ADL	2.49	4.78	9.62	5.75
PB	12.31	11.18	13.78	10.94
GB	5.74	6.75	4.51	5.86
**Fatty acids (g/100 g)**				
ΣSFA	15.27	12.88	16.28	11.91
ΣMUFA	23.80	59.32	41.71	56.69
ΣPUFA	22.25	9.18	25.11	7.27
n-6/n-3	17.18	7.77	14.08	7.57

DM—dry matter; OM—organic matter; NDF—neutral detergent fiber; ADF—acid detergent fiber; ADL: acid detergent lignin; PB—crude protein; GB—crude fat. C—control; BgCf15—Base diet + 15% olive cake two-phases; BgCf25—Base diet + 25% olive cake two-phases; and BgPr15—Base diet + 15% crude olive cake.

**Table 2:** Physicochemical composition of dry-cured Bísaro loin and dry-cured Bísaro “cachaço”.

Physicochemical Composition (g/100 g)																
	a_w_		Moisture		Ash		Total Fat		Protein		Collagen		Haem Pigments		NaCl	
	L	C	L	C	L	C	L	C	L	C	L	C	L	C	L	C
Control	0.893	0.857	41.27	31.24	5.07	4.97	21.40	41.09	32.61	24.52	3.44	3.15	2.31	4.17	3.75	4.24
Cf15	0.873	0.839	42.73	33.43	4.98	5.96	19.03	36.59	32.24	24.99	3.35	2.55	2.15	4.33	3.55	5.01
Cf 25	0.866	0.862	40.02	31.84	7.21	5.36	24.62	40.03	29.73	23.40	3.66	2.74	2.72	4.04	6.53	4.01
Pr15	0.896	0.815	41.69	30.99	4.25	6.13	16.90	43.37	34.26	24.39	2.83	2.87	2.41	3.84	2.76	5.36
SEM	0.01		1.37		0.57		3.46		1.13		0.56		0.29		0.53	
Significance product	***		***		ns		***		***		ns		***		ns	
Significance diet	ns		ns		ns		ns		ns		ns		ns		*	
Significance product x diet	*		ns		**		ns		ns		ns		ns		***	

ns—not significant, * *p* < 0.05; ** *p* < 0.01; *** *p* < 0.001. SEM (Standard Error of the Mean). L—dry-cured loin; C—dry-cured “cachaço”). Haem pigments in mg myoglobin/g fresh muscle. Cf15—Base diet + 15% olive cake centrifuged; Cf25—Base diet + 25% olive cake centrifuged; Pr15—Base diet + 15% olive cake pressed; CT—Base diet.

**Table 3:** Effect of diet in fatty acids profile in Bísaro dry-cured Loin and dry-cured “cachaço”.

Fatty Acids	L				C				SEM	*p*	D
	Control	Cf15	Cf25	Pr15	Control	Cf15	Cf25	Pr15			
C10:0	0.02	0.02	0.02	0.02	0.02	0.01	0.02	0.01	0.004	ns	ns
C12:0	0.05	0.05	0.05	0.05	0.04	0.05	0.04	0.05	0.003	ns	ns
C14:0	1.14	1.15	1.13	1.11	1.13	1.13	1.12	1.11	0.03	ns	ns
C14:1	0.03	0.03	0.03	0.02	0.02	0.03	0.03	0.02	0.005	ns	ns
C15:0	0.05	0.05	0.05	0.07	0.02	0.03	0.02	0.01	0.008	***	ns
C16:0	26.12	25.98	26.05	25.48	26.38	26.17	26.37	25.80	0.30	ns	ns
C16:1n-7	2.50	2.32	2.29	2.36	2.53	2.34	2.32	2.15	0.08	ns	**
C17:0	0.17	0.20	0.21	0.12	0.24	0.23	0.24	0.22	0.02	**	ns
C17:1n-7	0.23	0.22	0.22	0.19	0.24	0.23	0.23	0.20	0.02	ns	ns
C18:0	13.1	13.41	13.30	12.96	12.68	12.72	12.93	12.84	0.32	*	ns
9t-C18:1	0.21	0.23	0.19	0.21	0.20	0.22	0.18	0.22	0.01	ns	**
C18:1n-9	47.82	47.40	47.49	48.43	47.97	48.01	47.43	48.49	0.55	ns	ns
C18:2n-6	6.50	6.79	6.76	6.74	6.62	6.81	7.02	6.90	0.26	ns	ns
C20:0	0.21	0.19	0.21	0.22	0.17	0.17	0.18	0.18	0.01	***	ns
C18:3n-6	0.011	0.011	0.013	0.006	0.003	0.006	0.004	0.003	0.002	***	ns
C20:1n-9	0.77	0.80	0.91	0.82	0.76	0.80	0.86	0.79	0.03	ns	***
C18:3n-3	0.20	0.24	0.24	0.22	0.21	0.24	0.24	0.25	0.01	ns	***
C20:2n-6	0.24	0.24	0.2	0.25	0.23	0.24	0.28	0.24	0.01	ns	**
C22:0	0.04	0.05	0.03	0.05	0.03	0.04	0.03	0.03	0.03	*	ns
C20:3n-6	0.05	0.05	0.05	0.06	0.04	0.05	0.05	0.04	0.006	ns	ns
C22:1n-9	0.03	0.04	0.03	0.04	0.03	0.03	0.04	0.02	0.004	ns	ns
C23:0	0.35	0.40	0.33	0.43	0.27	0.32	0.22	0.29	0.05	***	ns
C24:1n-9	0.06	0.07	0.06	0.07	0.07	0.07	0.07	0.06	0.007	ns	ns
C22:6n-3	0.03	0.03	0.02	0.03	0.02	0.02	0.03	0.02	0.005	ns	ns
SFA	41.31	41.52	41.38	40.52	41.01	40.88	41.16	40.55	0.56	ns	ns
MUFA	51.65	51.11	51.23	52.15	51.83	51.73	51.15	51.95	0.56	ns	ns
PUFA	7.04	7.38	7.39	7.33	7.16	7.40	7.70	7.49	0.28	ns	ns
n-6	6.80	7.10	7.11	7.06	6.90	7.11	7.36	7.18	0.27	ns	ns
n-3	0.22	0.27	0.28	0.26	0.26	0.28	0.33	0.31	0.02	**	*
n-6/n-3	31.75	27.39	27.37	28.24	30.85	26.74	24.83	24.64	1.88	ns	**
IA	0.52	0.52	0.52	0.50	0.53	0.52	0.53	0.51	0.01	ns	ns
IT	1.35	1.36	1.35	1.30	1.33	1.32	1.34	1.3	0.03	ns	ns
h/H	2.00	2.01	2.01	2.09	2.00	2.02	2.00	2.07	0.04	ns	ns

ns—not significant, * *p* < 0.05; ** *p* < 0.01; *** *p* < 0.001. SEM (Standard Error of the Mean). Cf15—Base diet + 15% olive cake centrifuged; Cf25—Base diet + 25% olive cake centrifuged; Pr15—Base diet + 15% olive cake pressed; CT—Base diet. SFA, saturated fatty acids; MUFA, monounsaturated fatty acids; PUFA, polyunsaturated fatty acids; PUFA n-6/n-3 (∑ omega-6)/(∑ omega-3); IA, index of atherogenicity; IT, index of thrombogenicity; h/H = (C18:1n-9 + C18:2n-6 + C20:4n-6 + C18:3n-3 + C20; 5-n3 + C22:5n-3 + C22:6n-3)/C14:0 + C16:0; only fatty acids which represented more than 0.1% are presented in the table, although all detected fatty acids were used for calculating the totals and the indices.

## Data Availability

The original contributions presented in the study are included in the article, further inquiries can be directed to the corresponding author.
